# Regional innovation effect of smart city construction in China

**DOI:** 10.1371/journal.pone.0281862

**Published:** 2023-02-16

**Authors:** Yiwu Zeng, Zenghui Zhang, Zi Ye, Lili Li

**Affiliations:** 1 School of Economics, Hangzhou Normal University, Hangzhou, Zhejiang, China; 2 College of Economics and Management, Southwest University, Chongqing, China; 3 School of Public Management, Zhejiang University, Hangzhou, Zhejiang, China; 4 School of Economics, Hangzhou Dianzi University, Hangzhou, Zhejiang, China; Guangdong University of Foreign Studies, CHINA

## Abstract

Innovation is an important driving force for high-quality regional economic development. In recent years, the Chinese government has actively explored new ways to improve regional innovation level, and the construction of smart cities is regarded as an important measure to implement the innovation-driven development strategy. Based on the panel data of 287 prefecture-level cities in China from 2001 to 2019, this paper examined the impact of smart city construction on regional innovation. The research shows that: (i) The construction of smart city has significantly improved the level of regional innovation; (ii) Investment in science and technology and human capital level are important transmission paths for smart city construction to affect regional innovation; (iii) Compared with the central and western regions, the impact of smart city construction on regional innovation is more obvious in the eastern region. This study deepens the understanding of smart city construction, which has important policy significance for China to accelerate the construction of an innovative country and the healthy development of smart cities, and provides reference for other developing countries to promote smart city construction.

## Introduction

The global economy is still struggling to recover, deeply affected by many uncertain factors including the COVID-19 epidemic [1]. The economic development of developing countries is facing both opportunities and challenges. However, insisting on innovation-driven is the fundamental measure to seize opportunities and meet challenges as innovation is an important driving force for high-quality regional economic development [2]. Only by improving the level of regional innovation can developing countries promote the economic growth and industrial structure upgrading, and remain invincible in international competition. However, compared with developed countries, developing countries still have a long way to go in innovative development, which requires the government to constantly explore and overcome various difficulties.

China, as the largest developing country in the world, has actively explored new ways to promote regional innovation in recent years. Innovation activities are characterized by high complexity, high risk, and long cycle, and they cannot do without the support of the government [3,4]. Relevant studies show that government policies such as subsidies, industrial policies and financial support policies have an important positive impact on innovation [5,6]. Cities are the gathering places of various innovation elements and resources, and the revolution of urban development mode is of great significance to promote regional innovation. China attaches great importance to the construction of smart city as an important measure to implement the innovation-driven development strategy. Smart city is the product of the deep integration of informatization and urbanization, an advanced stage of urbanization, and a comprehensive innovation of urban development models. The construction of a smart city is the comprehensive application of new information technologies such as the Internet of Things, cloud computing, big data, artificial intelligence, and blockchain to achieve intelligent, efficient, fair, harmonious, green, livable and sustainable development in urban planning, construction, management, service and environment. In order to explore and promote the construction of smart city, China successively set up three batches of national smart city pilot projects in January 2013, August 2013 and April 2015, involving 299 cities (districts, counties and towns), and formulated a series of important documents to guide the implementation of pilot work. Although many developing countries are also advancing the construction of smart cities, China’s advancement is faster and wider.

Then, has the construction of smart cities in China significantly improved the level of regional innovation? What are the main transmission paths through which smart city construction influences regional innovation? Is there regional heterogeneity in the regional innovation effect of smart city construction? To address these issues, based on the panel data of 287 prefecture-level cities in China from 2001 to 2019, this paper uses two-way fixed effect model, mediating effect model and group regression method to empirically analyze the overall impact, main transmission paths and regional heterogeneity of smart city construction on regional innovation. The value of this paper is as follows: First, taking China as an example, it identifies the influence and mechanism of smart city construction on regional innovation, clarifies the important role of smart city construction in improving regional innovation level, and provides empirical evidence for promoting smart city practice in developing countries. Second, the economic and social effects of smart city construction are investigated from the perspective of regional innovation, which expands the research perspective of smart city construction. Third, based on empirical results, we put forward some valuable suggestions for China and other developing countries on how to better promote the construction of smart cities.

## Literature review

The predecessor of smart city is the smart comfort community proposed by Gore (1998) [7]. Since then, in order to better cope with the "urban diseases" such as traffic congestion, environmental deterioration and resource shortage, which are common in the process of global urbanization expansion, IBM first put forward the strategic concept of "smart earth" in 2008, and regarded "smart city" as a key part of this strategy. Smart city, a brand-new concept of urban construction and governance, soon received positive responses from developed countries such as the United States, the United Kingdom, Japan, Germany, the Netherlands, Singapore and South Korea [8], and then affected developing countries, which subsequently set off an upsurge of smart city construction.

Regarding the connotation of smart cities, scholars have defined it from different angles. From the perspective of economic growth, some scholars believe that smart cities refer to regions or cities that have recognized the importance of realizing the broadband economy and consciously take measures that can create vigorous economic development [9]. From the perspective of resources and environment, smart cities refer to more efficient, sustainable, fair and livable cities [10]. From the perspective of infrastructure, smart city refers to a city that uses smart computing technology to make urban infrastructure more intelligent, interconnected and effective [11]. From the perspective of urban governance, smart city refers to a city where citizens make independent, conscious, and their own decisions, and various stakeholders act intelligently and efficiently in an innovative and forward-looking way of management in terms of economy, manpower, supervision, transportation, environment and life. It provides citizens with advanced, user-centric and user-created services [12]. From the perspective of a socio-culture, a smart city is a city that can inspire inspiration and share culture, knowledge and life, a city that promotes the prosperity of residents’ lives, a desirable city, and an autonomous space full of knowledge [13].

As for the economic and social effects of smart city construction, the existing literature mainly focuses on these aspects: First is the impact of smart city construction on government management. In the construction of smart city, all parts of the city are integrated into an innovative urban ecosystem based on information technology, where meticulously designed processes and standardized formats are adopted between various government departments and between the government and other governance subjects [14]. Second is the impact of smart city construction on service supply. In the construction of smart city, the widespread application of information and communication technology reduces the cost and fragmentation of public service supply, and effectively improves the efficiency and accuracy of public services [15]. Smart cities can provide personalized services for citizens with diverse needs more efficiently [16]. Third is the impact of smart city construction on social capital. In the construction of smart city, the formation of social information network makes it possible to the promotion of social inclusion and the development of social capital [17]. Fourth is the impact of smart city construction on sustainability. The link between city smartness and carbon dioxide emissions is not linear, and the impact of city smartness on carbon dioxide emissions does not change over time [18]. This finding calls for better alignment of smart city strategies to achieve concrete sustainable outcomes. Fifth is the impact of smart city construction on citizen participation and subjective well-being. Granier & Kudo (2016) revealed the great potential of smart cities to promote civic engagement, using the example of smart communities in Japan [19]. Giffinger et al. (2010) believed that smart city could greatly improve the living comfort and happiness of residents [20].

In general, although smart city is still a novelty in theory and concept, it has become an ideal model of future urban development for urban planners and managers all over the world. Throughout the United States, Europe and developed countries in Asian including Japan, South Korea and Singapore, smart city construction projects are in full swing, and have achieved remarkable results [21]. However, there are few literatures that examine the economic and social effects of smart city construction in developing countries from the perspective of regional innovation. Therefore, this paper takes China as an example to study the impact of smart cities on regional innovation, which is of positive literature significance. China is an emerging market economy that is in the midst of large-scale smart city construction. The empirical evidence obtained can undoubtedly add important findings based on developing countries to the field of smart city research.

## Background and hypothesis

### Smart city construction in China

The first stage is the exploration period from 2010 to 2013. In 2010 and 2011, there were policy vacuums at the national level. In these two years, only individual local governments promoted the construction of smart cities according to their own understanding, which were relatively scattered and disorderly. In November 2012, Ministry of Housing and Urban-Rural Construction issued “the Notice on Carrying Out the National Smart City Pilot Work”, “the Interim Measures for the Administration of the National Smart City Pilot” and “the National Smart City (District, Town) Pilot Index System (Trial)”, marking China’s official exploration of smart city construction at the national level. In January and August 2013, Ministry of Housing and Urban-Rural Construction successively announced the first and second batch of National Smart City Pilot lists, and more cities have joined the ranks of smart city construction. The first batch consisted of 90 pilot projects and the second batch consisted of 112 pilot projects including 103 new pilots and 9 expansion pilots.

The second stage is the promotion period from 2014 to 2016. In October 2014, China established the "inter-Ministerial Coordination Working Group for the Healthy Development of Smart Cities", led by the Development and Reform Commission and composed of 25 ministries and commissions. All departments began to cooperate and guide local governments to carry out smart city construction. During this period, the central government also successively issued important documents such as “the National New Urbanization Plan (2014–2020)” and “the Guiding Opinions on Promoting the Healthy Development of Smart Cities”, vigorously guiding the construction of smart cities and setting off a wave of smart city construction by various ministries and municipalities. In April 2015, the Ministry of Housing and Urban-Rural Construction and the Ministry of Science and Technology jointly released the third batch of National Smart City pilot list, with a total of 97 pilots, including 84 new pilots and 13 expansion pilots. So far, there are 299 pilot cities (districts, counties and towns) for smart city construction in China.

The third stage is the promotion period from 2017 to now. In the process of building smart cities, there are problems such as deviation from the main line of development, repeated construction, waste of resources, inefficient operation and neglect of urban service targets in various regions [22], which deviates from the central government’s presupposition and original intention for smart city construction. In July 2016, the central government issued “the Outline of National Informatization Development Strategy”, proposing the construction of "new smart cities", and carrying out reasonable guidance, deviation correction and connotation improvement for the construction of smart cities around the country. Since then, policies concerning the construction of new smart cities have been issued one after another, such as "the Notice on Organizing and Carrying Out the Evaluation of New Smart Cities and Practically Promoting the Healthy and Rapid Development of New Smart Cities" issued in November of the same year, and “the Thirteenth Five-Year Plan" issued in December of the same year. The new smart city is an iterative evolution of smart city construction from version 1.0 to version 2.0. It emphasizes people-oriented, reform and innovation, and focuses on promoting technology integration, business integration and data integration to achieve cross-level, cross-regional, cross-system, cross-departmental and cross-business collaborative governance, and ultimately realize the improvement of urban development model and the improvement of urban quality [23].

From the first exploration in Ningbo, Shanghai and Nanjing and other cities, to the release of three batches of national smart city construction pilot lists, and then to the accelerated implementation of new smart city construction, China’s smart city construction is fast and extensive, leading the way among developing countries.

### Hypothesis

#### Overall impact of smart city construction on regional innovation

Reform and innovation is the essence of smart city construction, and it is necessary to pay attention to the long-term cultivation and continuous improvement of urban innovation ability. The significant improvement of regional innovation level is the inherent requirement of smart city construction, and also one of the important criteria to judge the success of smart city construction. With the promotion of smart city construction, the level of urban informatization is constantly improved, which is conducive to improving the level of regional innovation [24]. The construction of smart city is not only conducive to improving the utilization efficiency of information resources, so as to realize intelligent and refined urban management and services, but also to promote the development of emerging industries and the adjustment of industrial structure. The development of the Internet of things, cloud computing, big data, artificial intelligence, blockchain and other high-tech industries provides technical support for the development of innovation activities in all walks of life. High-tech industries are usually highly clustered, and industrial agglomeration can promote knowledge dissemination and technology spillover, and promote the formation of new ideas [25–28]. The construction of smart city promotes the development and application of cutting-edge information technologies such as Internet of Things, cloud computing, big data, artificial intelligence and blockchain, accelerates the information transmission and processing speed of urban enterprises, effectively reduces the information transmission cost, information asymmetry and uncertainty [29], and restrains opportunistic behaviors. Furthermore, it is conducive to improving the R&D efficiency and achievement transformation efficiency of enterprises and promoting the overall social innovation process. The information integration and data sharing promoted by the construction of smart cities enable innovation subjects to obtain more accurate information and data, and make more scientific and intelligent innovation decisions and management. The breaking of information barriers also helps all subjects to carry out higher levels of collaborative innovation and open innovation [30,31]. The construction of smart city also provides more potential opportunities for new products and extended services of technology or knowledge intensive enterprises, because the development of digital economy will induce urban residents’ demand for commodity consumption to become personalized, diversified and high-quality, opening the market space for "long-tail products" and promoting the growth of production and variety of "long-tail products" [32–34]. Smart cities build an institutional environment conducive to the emergence of innovation, and a superior business environment helps reduce the cost of innovation and improve the efficiency of enterprises [35]. With the improvement of urban institutional environment, enterprises will have more time and resources to allocate to innovation activities, thus improving their innovation capability [36].

Based on the above analysis, hypothesis 1 is proposed:

H1: Smart city construction has a positive effect on regional innovation.

#### The influence paths of smart city construction on regional innovation

Capital and manpower are the two major input factors of innovation activities. The improvement of regional innovation level cannot be achieved without large amount of financial support and high level of human capital guarantee [37,38]. Innovation has the characteristics of high investment, high risk and high return, and usually needs the support of public financial input from the government, especially in the aspects of innovation infrastructure, enterprise subsidies, and the development of science and technology insurance. The construction of smart city is a development process in which the government promotes the deep integration of informatization and urbanization in a planned way. The Chinese government has consciously arranged financial funds to promote the construction of smart cities. The construction of smart city is an important strategic measure, and the policy of the central government drives the financial expenditure of local governments at all levels in the field of science and technology. Investment in science and technology can provide necessary financial support for innovation activities, and then exert a positive influence on the innovation effect of smart cities. In addition, investment in science and technology will also drive the supporting investment and collaborative participation of social capital, and stimulate the development of the smart finance industry. The wisdom of financial products, risk control and services effectively reduces financing costs and risks, and encourages scientific and technological innovation subjects to participate in innovation practices, thus promoting the improvement of regional innovation capabilities [39]. Human capital is a form of capital that condenses on people and can be used in production or service activities, which is specifically reflected in the skills, knowledge and physical strength possessed by workers [40]. Smart city starts from human capital, and its core is people [41]. On the one hand, smart cities provide diversified opportunities for the development of human capital potential and the realization of creative life, which can greatly attract and retain talents and increase human capital at the quantitative level; On the other hand, the rigid demand of smart city for high-end human capital makes smart cities a gathering center of higher education and well-educated talents [42], which will improve human capital in terms of quality. The human capital improvement effect brought by smart cities in both quantity and quality is conducive to accelerating the spread, diffusion and spillover of knowledge [43] and promoting the improvement of regional innovation ability.

Based on the above analysis, hypothesis 2 is proposed:

H2: Investment in science and technology and human capital level are important transmission paths for smart city construction to affect regional innovation.

#### Heterogeneity of the impact of smart city construction on regional innovation

Smart city construction is a systematic, complex and long-term project based on the new generation of information technology, guaranteed by financial support, and driven by high-tech talents. Its specific connotation covers all aspects of urban system operation, including infrastructure from the bottom to the top of the public governance. Therefore, it needs a certain economic foundation, technical conditions, human capital, public finance, etc [11]. There are significant differences in basic resources, economic development level, institutional quality and other aspects in different regions of China, which may lead to obvious differences in the effect of the same degree of information investment on innovation capability in different regions. In other words, the impact of information investment on innovation capability in different regions may be heterogeneous. Compared with the central and western regions, cities in the eastern region have excellent urban resource endowment, high level of economic development, relatively perfect information infrastructure construction, good market operation environment, strong local financial strength and attract a large number of high-quality talents. With all these existing advantages, the construction of smart cities in the eastern region has been promoted at a fast pace, key smart applications have been in-depth construction, and the effects of some key application fields have begun to appear. Network operators, internet platform enterprises and electronic information equipment manufacturing enterprises are important subjects to promote the construction of smart cities. However, the strength and effectiveness of cooperation between central and western cities and these third-party organizations are far from reaching the level of eastern cities, and some have not yet introduced any third-party organization to carry out smart city operation and management [44]. With the continuous promotion of smart city construction, the economic and social effects of smart city construction will also be more fully released in the central and western regions. At present, the eastern region leads the country in the construction of smart cities, and its regional innovation effect will be more prominent.

Based on the above analysis, hypothesis 3 is proposed:

H3: The impact of smart city construction on regional innovation is regionally heterogeneous.

## Methods and data

### Estimation method

Based on the panel data of Chinese prefecture-level cities, this paper constructs the following two-way fixed effect model to test the impact of smart city construction on regional innovation:

$$RI_{i,t}=\alpha_{0}+\alpha_{1}SC_{i,t}+\alpha_{2}FE_{i,t}+\alpha_{3}HC_{i,t}+\alpha_{c}Z_{i,t}+\mu_{i}+\delta_{t}+\varepsilon_{i,t}$$ (1)

Where *i* represents prefecture-level city, *t* represents year, *RI*_*i*,*t*_ represents the regional innovation level, *Smartcity*_*i*,*t*_ is a dummy variable for the smart city pilot, *FE*_*i*,*t*_ represents the investment in science and technology, *HC*_*i*,*t*_ represents the human capital level, *Z*_*i*,*t*_ includes a series of control variables, *u*_*i*_ is the city fixed effect, *δ*_*t*_ is the time fixed effect, and *ε*_*i*,*t*_ is the random term.

This paper uses the mediating effect model to explore the mechanism of the influence of smart city construction on regional innovation, and specifically tests whether the two transmission paths of investment in science and technology and human capital level are valid. On the basis of Eq (1), this paper further constructs three regression equations in which smart city construction affects regional innovation (excluding investment in science and technology and human capital level), smart city construction affects investment in science and technology, and smart city construction affects human capital level as follows:

$$RI_{i,t}=\theta_{0}+\theta_{1}SC_{i,t}+\theta_{c}Z_{i,t}+\mu_{i}+\delta_{t}+\varepsilon_{i,t}$$ (2)

$$FE_{i,t}=\beta_{0}+\beta_{1}SC_{i,t}+\beta_{c}Z_{i,t}+\mu_{i}+\delta_{t}+\varepsilon_{i,t}$$ (3)

$$HC_{i,t}=\gamma_{0}+\gamma_{1}SC_{i,t}+\gamma_{c}Z_{i,t}+\mu_{i}+\delta_{t}+\varepsilon_{i,t}$$ (4)

If the coefficient of *θ*_1_ is significant, further observe the significance level of the coefficients of *β*_1_, *γ*_1_, *α*_2_ and *α*_3_; Otherwise, the mediating effect test ends. On the basis of significant coefficient of *θ*_1_, if both *β*_1_ and *α*_2_ are significant, it means that at least part of the influence of smart city construction on regional innovation is transmitted through the mediating variable of investment in science and technology. If both *γ*_1_ and *α*_3_ are significant, it means that at least part of the impact of smart city construction on regional innovation is transmitted through the intermediary variable of human capital level. Then if *α*_1_ is not significant, it indicates that the influence of smart city construction on regional innovation is completely affected by mediating variables. If *α*_1_ is significant, it indicates that the financial expenditure of science and technology and the level of human capital only play a part of the mediating role.

In order to test whether the impact of smart city construction on regional innovation has regional heterogeneity, this paper divided all the samples into three groups: the eastern region sample, the central region sample and the western region sample for regression, and compared the regression coefficient and significance level of the core explanatory variables in the regression results of the three groups.

### Variables

The dependent variable is Regional innovation, which is measured by the number of patent applications granted per 10,000 people. Some scholars advocate the use of new product scale to reflect the level of regional innovation, but in China, new products are not uniformly defined, measured and statistically monitored. Some scholars use the amount of science and technology input and the number of patent applications received to measure the level of regional innovation. However, compared with the amount of science and technology input and the number of patent applications received, the amount of patent applications granted can more directly and accurately reflect the level of regional innovation. Although science and technology input is the basis of science and technology output, science and technology input does not directly reflect science and technology strength, because input and output may not be equivalent. Although the number of patent applications received reflects the innovation vitality of a region, patent applications do not necessarily pass the review and obtain final authorization, so it can not accurately reflect the strength of technological innovation. Some scholars tend to use the number of patent applications because patent licensing needs to pay an annual fee, and they think that the data is unstable and not updated in time. But in fact, because the cost of patent application is not large, a large number of patent applicants are holding the trial and luck psychology, and the result is that the success rate of obtaining authorization is low. Therefore, using the number of patent applications is easy to overestimate the level of regional innovation. In addition, annual data are used in this paper, which does not have the problem of data instability and untimely update. Therefore, this paper uses the number of patent applications granted to measure the level of regional innovation. Considering the differences in population size in different regions, the number of patent applications granted per 10,000 people is adopted by dividing the number of patent applications granted by the total population at the end of the year.

The independent variable is Smart city construction, measured by a dummy variable of whether it is listed as a smart city pilot. The prefecture-level city including a smart city pilot in the current year is assigned as 1, and the remaining prefecture-level city is assigned as 0.

The mediating variables are Investment in science and technology and Human capital level. Combined with data availability, the proportion of science budget expenditure in GDP and the number of students in colleges and universities per 100 people are respectively used to measure. Budget expenditure, also known as purchasing fiscal expenditure, refers to the process of allocating funds collected from the state budget in a planned manner according to certain methods and channels, which is the guarantee of financial resources for the realization of government functions. It is an important link of financial distribution activities, which reflects the national policy and stipulates the scope and direction of government activities. College students have a higher level of human capital and are the dominant group in the labor market. The more college students a region has, the more advanced its higher education is. Because education is the most important investment channel of human capital, it is a common practice for Chinese scholars to use the proportion of university students to measure the level of human capital in a region.

Drawing on the relevant research on innovation [23,37,38,45–47], this paper also includes other control variables: Economic development is expressed by the logarithm of GDP per capita; Industrial structure is measured by dividing the proportion of the added value of the secondary industry in GDP by the proportion of the added value of the tertiary industry in GDP; Population size is expressed by the logarithm of total registered population at the end of the year; Opening degree is measured by the proportion of import and export value in GDP; Urban employment is measured by the proportion of the number of employees in urban units in registered population at the end of the year. Generally, the higher the level of economic development, industrial structure, population size, openness and urban employment, the higher the level of regional innovation.

**Fig 1:**
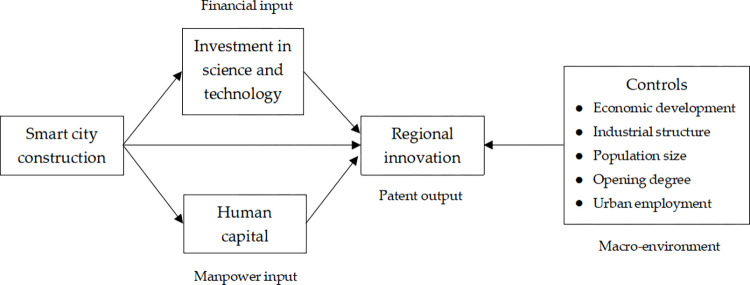
Empirical framework for measuring the impact of smart city construction on regional innovation.

In summary, this paper constructs an empirical framework as shown in Fig 1 to measure the impact of smart city construction on regional innovation. On the one hand, smart city construction has a direct effect on regional innovation. On the other hand, smart city construction has an indirect effect on regional innovation by affecting the level of science and technology investment and human capital. These two transmission paths represent the two key input factors of innovation financial resources and innovation manpower respectively. In other words, this paper constructs the empirical framework of influencing mechanism from the perspective of innovation input. Correspondingly, the measure of regional innovation level uses patent output. In addition, to avoid the omission bias problem, we add control variables reflecting the economy, population, opening to the outside world, industry, and employment, which are macro environmental factors affecting innovation, into the estimation equation.

### Data

The paper uses panel data from 287 prefecture-level cities in China from 2001 to 2019. The dummy variable of smart city pilot is based on the list of three batches of national smart city pilots published on the official website of the Ministry of Housing and Urban-Rural Construction of the People’s Republic of China. As there may be one or more pilots in a prefecture-level city, the 299 smart city pilots established do not correspond to 299 prefecture-level cities. Moreover, prefecture-level cities such as Laiwu and Chaohu that have undergone administrative division adjustment during the sample investigation period are eliminated; cities such as Bijie and Puer that have undergone administrative division adjustment or city names have changed below the prefecture level are retained; prefecture-level cities such as Sansha and Danzhou where statistical data are seriously missing are excluded. After screening, a total of 165 prefecture-level cities constitute the "experimental group", while the remaining 122 prefecture-level cities that are not included in the smart city pilot list enter the "control group". The regional innovation level is measured by the number of patent applications granted per 10,000 people, which comes from Chinese Research Data Services (CNRDS). The basic data of other variables are from "China City Statistical Yearbook", "China Regional Economic Statistical Yearbook", statistical yearbooks of prefecture-level cities, and National Economic and Social Development Statistical Bulletins over the years. The basic definition and statistical characteristics of each variable are shown in Table 1.

**Table 1:** Definition and descriptive statistics of the variables.

Variable Name	Definition	Mean	S.D.
*Dependent variable*			
Regional innovation	the number of patent applications granted per 10,000 people	6.060	17.246
*Independent variable*			
Smart city construction	whether it is listed as a smart city pilot, yes = 1, no = 0	0.201	0.401
*Control variable*			
Investment in science and technology	the proportion of scientific fiscal budget expenditure in GDP ratio	0.202	0.283
Human capital	the number of students in colleges and universities per 100 people	1.523	2.143
Economic development	the logarithm of GDP per capita	0.875	0.918
Industrial structure	proportion of added value of secondary industry in GDP/proportion of added value of tertiary industry in GDP	1.352	0.753
Population size	the logarithm of total registered population at the end of the year (10,000 persons)	5.853	0.701
Opening degree	the proportion of import and export value (thousands of US dollars) in GDP (10,000 yuan)	0.304	0.548
Urban employment	the proportion of the number of employees in urban units in registered population at the end of the year	0.119	0.122

## Results

### Baseline regression

Table 2 presents the baseline regression results. Columns (1) and (2) only take the core explanatory variables of smart city construction in the regressions. Columns (3) and (4) further include all control variables. Standard errors are clustered to the prefecture-level city level in columns (1) and (3), while the standard errors are clustered to the provincial level in columns (2) and (4) to control the correlation of error items that may exist between prefecture-level cities in the same province. The results of all regressions show that the regression coefficient of smart city construction on regional innovation is significantly positive at the confidence level of 5% or 1%, indicating that smart city construction promotes the improvement of regional innovation. So hypothesis 1 is validated. In columns (3) and (4), the regression coefficient is about 2.677, which means that the construction of smart city pilot projects in China has made the number of patents applications granted per 10,000 people in prefecture-level cities of the experimental group is 2.677 more than that of prefecture-level cities of the control group without the smart city pilot.

**Table 2:** The baseline estimation result.

Variables	Regional innovation			
	(1)	(2)	(3)	(4)
Smart city construction	7.102*** (1.747)	7.102** (3.041)	2.677*** (1.035)	2.677*** (0.959)
Investment in science and technology	— —	— —	9.973*** (2.883)	9.973** (3.923)
Human capital	— —	— —	1.542* (0.835)	1.542* (0.832)
Economic development	— —	— —	10.223*** (2.041)	10.223** (3.752)
Industrial structure	— —	— —	1.265** (0.622)	1.265 (0.957)
Population size	— —	— —	48.139** (22.951)	48.139 (33.963)
Opening degree	— —	— —	11.107** (4.675)	11.107* (5.897)
Urban employment	— —	— —	38.365* (20.850)	38.365 (22.660)
Constant	— —	— —	-286.408** (133.354)	-286.408*** (198.261)
City fixed effect	YES	YES	YES	YES
Year fixed effect	YES	YES	YES	YES
R2 _within	0.215	0.215	0.476	0.476
Number of observation	5,431	5,431	4,514	4,514

Standard errors are clustered to the prefecture-level city level in columns (1) and (3), while standard errors are clustered to the provincial level in columns (2) and (4).

Standard errors are reported in parentheses.

*, ** and *** denote statistical significance at 10%, 5%, and 1% levels, respectively.

### Transmission pathway

Table 3 reports the test results of the transmission path of smart city construction affecting regional innovation. Column (5) examines the impact of smart city construction on regional innovation without the variable of investment in science and technology, and column (6) examines the impact of smart city construction on investment in science and technology. Column (7) examines the impact of smart city construction on regional innovation without the variable of human capital, and column (8) examines the impact of smart city construction on human capital. Based on all estimated results of Table 3 and the estimated result of column (4) in Table 2, we can conclude that investment in science and technology and human capital play a partial intermediary effect in the positive relationship between smart city construction and regional innovation. In other words, investment in science and technology and the improvement of human capital levels are important transmission paths for smart city construction to affect regional innovation. So hypothesis 2 is validated.

**Table 3:** Transmission pathway test.

Variables	Regional innovation	Investment in science and technology	Regional innovation	Human capital
	(5)	(6)	(7)	(8)
Smart city construction	3.368*** (1.075)	0.073** (0.027)	3.020*** (0.952)	0.208** (0.094)
Investment in science and technology	— —	— —	10.197** (4.006)	— —
Human capital	1.676* (0.829)	— —	— —	— —
Other variables	YES	YES	YES	YES
City fixed effect	YES	YES	YES	YES
Year fixed effect	YES	YES	YES	YES
R2 _within	0.442	0.423	0.469	0.297
Number of observation	4,523	4,515	4,572	4,515

Standard errors are clustered to the provincial level.

Standard errors are reported in parentheses.

*, ** and *** denote statistical significance at 10%, 5%, and 1% levels, respectively.

### Heterogeneous effects

China’s regional economic development is unbalanced, and the level of economic development is decreasing from the east coast to the inland regions. Due to the differences in resource endowment, geographical location, economic development stage, humanities and history, there are significant differences in the construction level of smart city in each region. The smart city construction in eastern region is earlier and at a higher level with a strong comprehensive strength, followed by the central region, and finally the western region. The impact of smart city construction on regional innovation is likely to be unbalanced at the regional level due to the level of smart city construction. Table 4 presents the sub-regional regression results of the impact of smart city construction on regional innovation. We can see that the construction of smart cities in the eastern region has the largest impact on regional innovation, followed by the central region, and finally the western region. In addition, the driving effect of smart city construction on regional innovation in western China is not significant. This indicates that, compared with the central and western regions, the construction of smart cities in the eastern region has a more obvious impact on regional innovation, and hypothesis 3 is verified. This empirical result shows that there is also a digital divide phenomenon in the regional dimension of smart city construction. There is no denying that the construction of smart cities will benefit many people, but regional differences in digital development should also be concerned. Regional inequality is a common problem in developing countries, and smart city construction does not seem to play a role in narrowing the regional gap, but continues the original trajectory.

**Table 4:** The sub-regional regression results of the impact of smart city construction on regional innovation.

**Variables**	**Regional innovation**		
	**Eastern region**	**Central region**	**Western region**
	**(9)**	**(10)**	**(11)**
Smart city construction	5.472* (2.495)	1.149** (0.419)	0.167 (1.208)
Other variables	YES	YES	YES
City fixed effect	YES	YES	YES
Year fixed effect	YES	YES	YES
R2 _within	0.690	0.646	0.559
Number of observation	1,818	1,729	967

Standard errors are clustered to the provincial level.

Standard errors are reported in parentheses.

*, ** and *** denote statistical significance at 10%, 5%, and 1% levels, respectively.

### Robust checks

#### Placebo test

**Fig 2:**
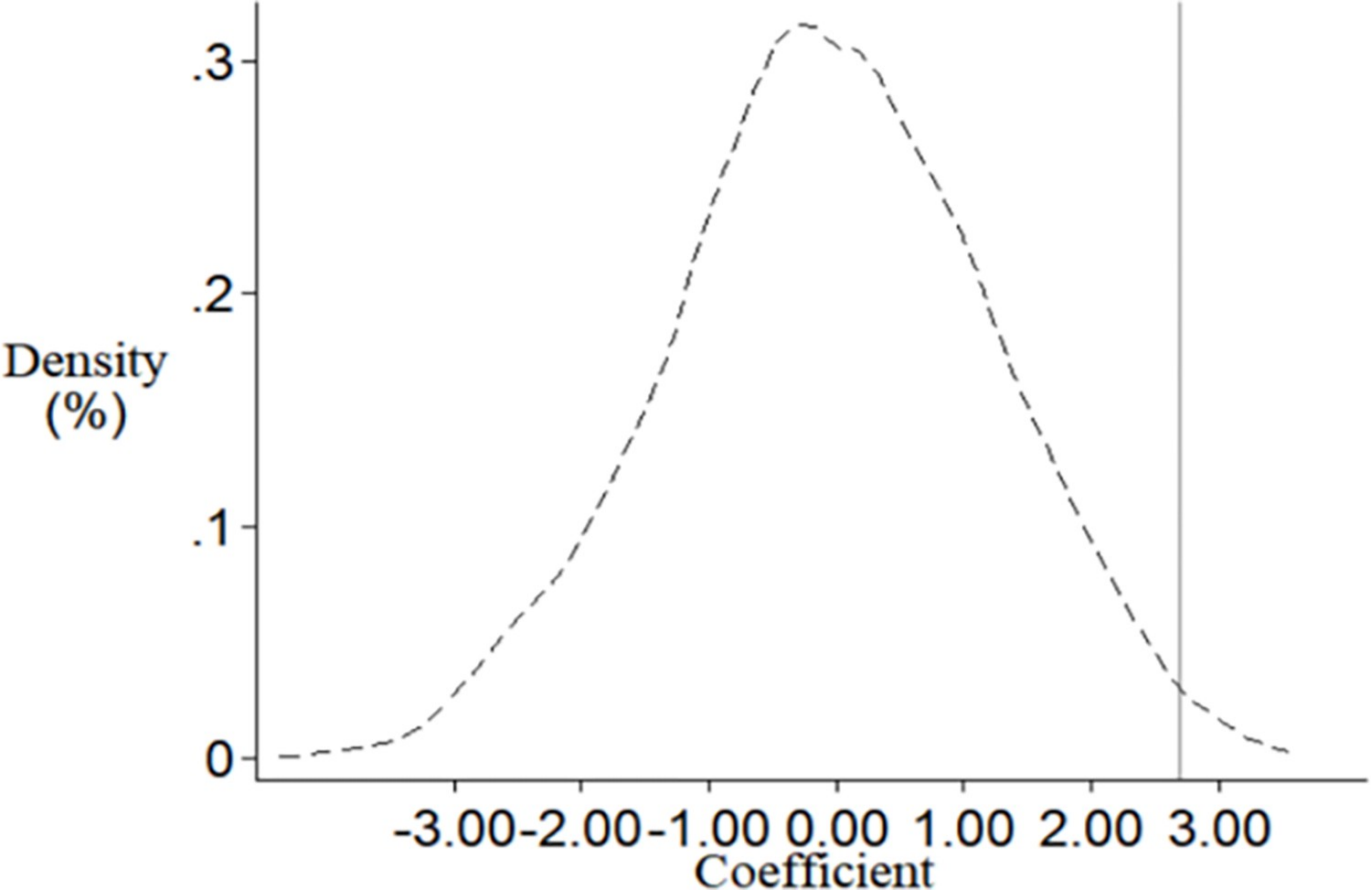
Placebo test based on randomly generated experimental sample.

This paper also draws on Li et al. (2016) and Cantoni et al. (2017) to randomly generate counterfactual samples of smart city pilots to conduct a placebo test to determine whether the significant impact of smart city construction on regional innovation is caused by some random factors [48,49]. Specifically, 165 prefecture-level cities were randomly selected from all samples in the paper, which were set as "pseudo experimental group" (the distribution of selected pilot cities in each year was also consistent with the real situation), while the remaining prefecture-level cities were included in the control group, so as to construct a "pseudo dummy variable" for regression. This paper repeats the above random generation process 1,000 times, and plots the distribution of the estimated coefficients of the randomly generated experimental group. As shown in Fig 2, the coefficients obtained by placebo test only rarely exceed the true regression value of 2.677, and the whole shows a normal distribution centered on 0. This shows that the result of smart city construction promoting regional innovation is not caused by other unobservable factors, and the baseline regression results have no obvious omitted variable bias.

#### Changing the measurement

This paper also changes the measurement index of regional innovation level to conduct a robust test to improve the reliability of the research conclusions. Specifically, this paper tried the urban innovation index. Unlike the number of patent applications granted, which only considers the number of patents obtained in the current year, the urban innovation index is a stock index that adjusts the value of patents. It not only measures intangible capital stock by patent value, but also distinguishes the average value of patents at different ages. By adding up the value of patents at different ages, the total intangible capital stock can be obtained, so it can measure the regional innovation level more accurately. The data for the urban innovation index comes from the "Report on China’s Urban and Industrial Innovation Power 2017" issued by the Industrial Development Research Center of Fudan University. Unfortunately, the time frame of the available data is limited to 2001–2016, which will lead to a significant decline in the size of observation samples and the timeliness of the data. Table 5 presents the regression results after changing the measurement. Column (12) is a full-sample analysis, and columns (13), (14) and (15) are sub-sample analysis of eastern, central and western regions, respectively. The estimated results all show that even if the measurement indicators are replaced, the results of smart city construction affecting regional innovation are still robust.

**Table 5:** The regression results of regional innovation measured by urban innovation index.

Variable	Regional innovation: Urban innovation index			
	Full sample	Eastern region	Central region	Western region
	(12)	(13)	(14)	(15)
Smart city construction	11.644** (4.387)	28.873* (13.216)	3.171*** (0.953)	0.409 (4.417)
Other variables	YES	YES	YES	YES
City fixed effect	YES	YES	YES	YES
Year fixed effect	YES	YES	YES	YES
R2 _within	0.236	0.376	0.365	0.259
Number of observation	3659	1477	1403	779

Standard errors are reported in parentheses. Standard errors are clustered to the provincial level.

*, ** and *** denote statistical significance at 10%, 5%, and 1% levels, respectively.

#### Eliminating extreme values

In order to prevent some extreme values from interfering with the estimation results, we conducted three tail-tails of 1%, 5% and 10% respectively for the number of patent applications granted per 10,000 people, and re-estimated the baseline model. It can be seen from Table 6 that even if the sample is curtailed, the construction of smart cities still significantly positively affects regional innovation, which further strengthens the research conclusions of this paper.

**Table 6:** Regression results after tail reduction of sample.

Variable	Regional innovation		
	1%	5%	10%
	(16)	(17)	(18)
Smart city construction	2.812*** (0.976)	1.534*** (0.548)	0.870** (0.362)
Other variables	YES	YES	YES
City fixed effect	YES	YES	YES
Year fixed effect	YES	YES	YES
R2 _within	0.462	0.533	0.615
Number of observation	4,514	4,514	4,514

Standard errors are reported in parentheses. Standard errors are clustered to the provincial level.

*, ** and *** denote statistical significance at 10%, 5%, and 1% levels, respectively.

## Conclusion and implications

Enhancing regional innovation capacity and building an innovative city are the key to building an innovative country. With the continuous advancement of smart city construction, digital technology has become an unprecedented driving force to reshape the economy and society, opening up a new era of urban development. Smart city construction provides a great opportunity for developing countries to drive innovative development, and China is leading this trend. Since the 21st century, China has vigorously promoted the construction of urban information infrastructure and mobile network services. Smart city technology applications such as e-commerce, social learning, online travel and express takeout have been popularized in China. Many new technologies have been integrated into the daily life of urban residents, forming more diverse new business forms and new ecology. Emerging new industries such as short video, internet celebrity economy and sharing economy in smart cities are showing great vitality. Internet celebrity cities, online celebrity attractions, and online celebrity foods grab people’s attention from time to time, bringing unexpected resources and opportunities to local development. Based on the literature review and the background of China’s smart city construction, this paper proposes three research hypotheses, and empirically analyzes the overall impact, main transmission paths and heterogeneity of smart city construction on regional innovation based on the panel data of 287 prefecture-level cities in China from 2001 to 2019. The regression results show that the construction of smart city has significantly improved the level of regional innovation, and investment in science and technology and human capital level are important transmission paths for smart city construction to affect regional innovation. This result not only affirms the effect of China’s smart city construction, but also injects a booster to the developing countries. In the context of the slowdown of global economic growth, the global innovation field still shows a dynamic development trend, and the growth of innovation is higher than the economic development rate. Compared with some developed countries, China, as a developing country, has maintained a relatively high level of investment in innovation and scientific research, which is very beneficial to economic and social development. As a brand-new model of modern city operation and governance, smart cities have become a key point to drive regional innovation and lead China into the forefront of innovative countries. The empirical evidence from China shows the significant role of smart city construction in promoting regional innovation, which helps to strengthen the belief and determination of developing countries to firmly promote the construction of smart cities, and further consolidate people’s understanding that informatization positively affect regional innovation.

Smart city is a new mode of urbanization and a new stage of information technology, which will become an effective carrier to promote regional innovation in developing countries. To further strengthen the positive impact of smart cities on urban innovation, developing countries must fully release the catalytic effect of scientific fiscal expenditure and human capital on smart cities to promote urban innovation. In other words, China’s smart city construction can obtain good innovation dividends, thanks to the strong financial investment of governments at all levels around the pilot policy, and constantly improving the quality of human capital, especially increasing the stock of high-end human capital, so as to provide sufficient talent support for smart cities to drive urban innovation. The development of high and new digital technologies often requires a huge amount of capital investment to realize technological innovation, and the breakthrough of major basic technologies requires large-scale basic research, and the research of basic science has the attribute of public goods, so it becomes the responsibility of the government to undertake or fund it. With the promotion of smart city pilot policies, the increase of government investment in science and technology can build an important guarantee for regional innovation. On the one hand, government investment in science and technology can effectively strengthen the regional science and technology foundation, improve the allocation of factors, integrate social innovation resources, and provide resources for technological innovation. On the other hand, sufficient investment in science and technology provides an important material guarantee for attracting high-end talents, high-quality capital and the aggregation of advanced technologies. Smart city pilot policies stimulate local governments to increase investment in science and technology, promote the aggregation of various innovation factors, make the city produce continuous innovation results, so as to effectively improve the level of urban innovation. From China’s experience, the financial investment in smart city construction is mainly used to improve infrastructure and popularization of the new generation of information technology, guide and subsidize emerging industrial entities, accelerate the marketization process, reduce institutional transaction costs in the process of economic development, optimize the institutional business environment, and provide a free, comfortable, open and inclusive environment for innovative R&D activities, so as to accelerate the promotion effect of smart city on urban innovation. Since the full release of the regional innovation effect of smart city construction needs the assistance of such elements as human and property, it requires the governments of developing countries to further improve the level of urban human capital, increase the financial expenditure on science and technology, and attract more innovative talents in the process of building smart cities.

Although smart city construction has significantly promoted regional innovation in China, the process of smart city construction is not smooth, nor without weaknesses and deficiencies. Compared with the central and western regions, the impact of smart city construction in the eastern China on regional innovation is more obvious. Compared with the central and western inland regions, the eastern coastal regions have a higher degree of opening up and a stronger advantage of foreign investment, which accelerates the docking of urban innovation resources and capital and stimulates urban scientific and technological innovation. Convenient transportation conditions also further promote the agglomeration of factors and talents, accelerate economic development and stimulate innovation ability. There is still a lot of room for smart city development in China. In particular, China needs to address the weakness of regional differences. The gap between cities and towns should not become wider because of smart city development. The problem of regional digital divide is universal and difficult to manage [50]. Under market economy regulation, digital divide is inevitable phenomenon. Due to the differences in endowments of production factors among market competition subjects, the strong can deprive the weak through market rules. With economic growth, production factors are further concentrated in the strong, and the mobility and competition of factors aggravate the inequality of social wealth distribution. In the digital age, the Matthew effect of market regulation still exists. From the beginning, digital technology was not invented and created for the bottom groups of society. The regulation of market mechanism emphasizes free competition and survival of the fittest to find the optimal efficiency, so the inevitable result is the digital divide. Technological change is born with a screening mechanism. From ancient humans to modern society, technological change always creates a series of new technological thresholds, dividing the population into two groups. Those who cross the threshold can benefit from technological change, while those who fail to cross the threshold will suffer from technological change [51]. At present, digital technology is exacerbating the trend of inequality between groups and regions. The government cannot leave the release of digital dividends to the market, which will only love the rich rather than help the poor and the weak, and relying on social welfare is just a drop in the ocean. Bridging the digital divide will inevitably depend on government public policy. Strong government investments and measures are needed to bridge the digital divide within groups and between regions. More public resources should be given to backward areas and vulnerable groups. But in reality, the government may not do this very well. In the process of promoting smart city construction, the government may have uneven distribution of public resources, which exacerbates the digital divide between regions and groups. Whether a region can be established as a smart city pilot is affected by various factors such as its own development foundation, social network, and political resources. Areas with a better foundation are more likely to become pilots, and those with a low starting point and a lack of funding capacity often struggle to win bids and access public resources. All in all, the smart city offers many benefits for many people and makes many people’s life comfortable; however, the smart city can also be a divider of the society, because of the market’s regulating role and the government’s unbalanced allocation of public resources.

## Supporting information

S1 Datasheet (XLS)
